# Spatial and intraseasonal variation in changing susceptibility to extreme heat in the United States

**DOI:** 10.1097/EE9.0000000000000136

**Published:** 2021-02-26

**Authors:** Keith R. Spangler, Gregory A. Wellenius

**Affiliations:** a Boston University School of Public Health, Department of Environmental Health, Boston, Massachusetts;; b Brown University Department of Earth, Environmental, and Planetary Sciences, Providence, Rhode Island;; c Brown University School of Public Health, Department of Epidemiology, Providence, Rhode Island; and; d Institute at Brown for Environment and Society, Brown University, Providence, Rhode Island

## Abstract

Exposure to excessive heat is associated with a higher risk of death. Although the relative risk of death on extreme-heat days has decreased over the past several decades in the United States, the drivers of this decline have not been fully characterized. In particular, while extreme heat earlier in the warm season has been shown to confer greater risk of mortality than exposure later in the season, it is unknown whether this within-season variability in susceptibility has changed over time and whether it is modified by region, climatic changes, or social vulnerability.

**Methods:**

We used distributed-lag nonlinear models and meta-regression to estimate the association between ambient maximum daily temperature during the early, late, and overall warm seasons and the relative risk of mortality for two decades, 1973–1982 and 1997–2006, in 186 metropolitan areas in the United States. We assessed changes in relative risk nationally, regionally, and between places with differential changes in early-season relative extreme heat and indicators of social vulnerability.

**Results:**

Most of the reduction in heat-related mortality nationally between the two decades is driven by decreases in late-season mortality, while substantial early-season risk remains. This difference is most apparent in the Northeast, in cities with greater increases in early-season relative extreme heat, and in places that have become more socially vulnerable.

**Conclusions:**

Early-season heat mortality risks have persisted despite overall adaptations, particularly in places with greater warming and increasing social vulnerability. Interventions to reduce heat mortality may need to consider greater applicability to the early warm season.

## Abstract 2

Supplemental Digital Content is available in the text.

**What this study adds**

This study contributes important nuance to the assessment of long-term changes in heat-related mortality (i.e., susceptibility) by analyzing the early and late warm seasons separately. By narrowing the time period assessed, we demonstrate that while susceptibility to extreme heat in the late warm season and overall has decreased, considerable risk of heat-related deaths remains in the early warm season. Our results further suggest that both climatic and sociodemographic changes may contribute to remaining heat-health risks in the United States.

It is well documented that moderate and extreme heat are associated with human morbidity (illness) and mortality (death) in the United States.^1^ The public health impact of this is substantial: in the United States, more deaths are attributed to heat waves than to any other natural disaster,^2^ and the total public health burden of heat is likely to be vastly greater than the number of deaths directly attributed to extreme heat events.^3^

Several studies have found that heat-related deaths have declined overall in the United States in recent decades.^4–6^ Specifically, these studies suggest that population average susceptibility to heat has decreased, such that days of extreme heat today are associated with smaller relative risks (RRs) for death compared to RRs over the past several decades. However, the drivers of this decline in susceptibility to extreme heat are not fully understood. Although the proliferation of air conditioning (AC) systems is often cited as a likely contributor,^7^ one analysis did not find it to be a statistically significant effect modifier,^6^ and a more-recent article found that only about one-sixth of the reduction in heat-related mortality in the United States nationally is attributable to increased AC infrastructure in homes.^8^ Others have suggested that, in some places, improvements in population health, reductions in social vulnerability (SV), and heat-awareness campaigns may also play roles in declining heat susceptibility.^7,9–11^ The literature is not conclusive on the extent to which these adaptations have contributed to the reductions in heat susceptibility,^12^ though it is plausible that each plays a role to a varying degree depending on the geographic and sociodemographic context.^7^

In parallel, a few studies suggest the presence of intra-annual variability in heat susceptibility such that the RR of death on days of extreme heat is greatest earlier in the warm season. For example, Lee et al.^13^ found that temperature extremes relative to the time of year are associated with greater RRs of mortality, and Gasparrini et al.^14^ similarly showed that recent extreme-heat risks are statistically significantly greater in the first month of the warm season compared to the last. However, previous studies have not evaluated to what extent this within-season variability in heat susceptibility has changed over time. This is an important gap in the current knowledge because some of the posited drivers of reduced heat mortality—such as AC use or heat-warning systems—may be inconsistently utilized throughout the warm season, hence limiting their effectiveness. Moreover, while Gasparrini et al.^14^ showed that differences in within-season heat susceptibility vary between countries, it remains unknown how these differences manifest at regional scales within countries, which is crucial given the local nature of most adaptive measures. And while the relation between extreme heat and death is modified both by the local climate (higher RR in cooler places^15^) and sociodemographic characteristics (higher RR among the socially vulnerable, e.g., the elderly and those living in poverty^16,17^), it is not known whether changes over time in one or both of these components have been associated with temporal trends in heat mortality, either for the overall warm season or by the timing within the warm season. This is particularly important because recent trends in summertime warming in the United States may be disproportionately greater in places with more SV.^18^

To address these research gaps, we linked data on weather and more than 10 million deaths occurring over two decadal periods in 186 US cities. Specifically, we sought to evaluate whether the observed decline in heat susceptibility across the United States differed in the early versus late warm season, and spatially across regions of the United States. We further evaluated whether declines in heat susceptibility differed across strata of population-level markers of SV and climatic changes in early-season relative extreme heat (REH).

## Methods

### Data sources

#### Mortality data

**Figure 1.**
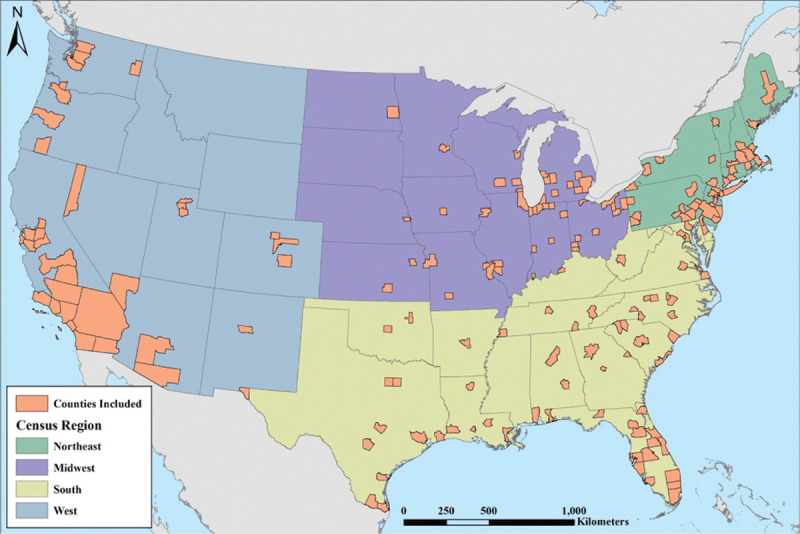
Map showing the distribution of the 186 metropolitan areas (defined by one or more counties) included in the analysis (orange) distributed across the four Census regions (Northeast, Midwest, South, and West). Background mapping is provided by ArcWorld and ArcWorld Supplement from Esri.

We obtained daily counts of all-cause mortality (for all ages, excluding external causes) from the National Center for Health Statistics for each of 186 metropolitan areas in the conterminous United States (Figure 1) for the period from 1973 to 2006, the latest publicly available data including both date of death and county of residence. These areas are defined by one or more counties due to the aggregation of mortality data, but we nonetheless refer to them hereafter as “cities.” Collectively, these areas accounted for approximately 56% of the total US population in 2000.^19^ Of the 186 cities included in the analysis, five of them (2.7%) included a county in which a boundary change occurred at some point during the study period; we assumed that this introduced a negligible amount of nondifferential error to the analysis and note that the time series modeling used is robust to changes in population size and characteristics that occur over the timescales analyzed. To assess changes in population average susceptibility to heat-related mortality over time, we divided the data into two decades (1973–1982 and 1997–2006) and subset to the city-specific warm season (interchangeably referred to hereafter as “summer”), which we defined as the five warmest months of the year: May to September in most locations, but June to October in some cities in Southern California. Two cities experienced shifts in the five warmest months between the two decades (San Jose, California and Riverside, California); to maintain consistency, we defined the warm seasons for these two cities using the five warmest months in the first decade. We chose to use only the first and last decade of available data to simplify the analysis and to more clearly identify the dominant long-term changes in heat susceptibility.

#### Climate data

We obtained daily maximum ambient temperatures (*T_max_*) from the TopoWx gridded meteorologic dataset^20^ for the period of interest (1972–2006). We used spatially resolved temperature estimates because our geographic units of analysis covered large areal extents, which may not adequately be represented by observations at a single weather station.^21,22^ We chose to use *T_max_* instead of *T*_*mean*_ primarily to maximize the applicability of the findings to policy interventions—particularly heat alerts, which are generally issued based on forecasted high temperatures or heat indices. To achieve spatial consistency with the metropolitan-scale mortality data, we calculated population-weighted averages of the *T_max_* estimates for each county based on the census tract population centroids from the 2000 Census:^23^ we extracted the TopoWx pixel value underlying each census tract centroid in the county of interest and weighted it by the proportion of the county population living in that tract. We then subset the *T_max_* values to the warm seasons of each city for 1973–1982 and 1997–2006 to align with the mortality data.

#### Sociodemographic data

We obtained county-level sociodemographic characteristics for the two decades of interest from the US Census Bureau’s 1980 Census and 2000 Census, respectively, via the IPUMS NHGIS database.^24^ We selected variables reflecting population proportions of: (1) persons over age 65 years living alone; (2) persons with income below the federal poverty line; (3) employees working in the agricultural or construction sectors; and (4) persons with a self-identified work disability. We selected these variables as population-scale indicators of potential susceptibility to heat,^7^ including elderly social isolation, lack of resources, all-day exposures to ambient temperatures, and preexisting medical conditions, respectively.

### Calculating additional metrics

We also calculated interdecadal changes in REH and SV to assess differences in heat-mortality relationships that are independent of geographic location or baseline climate (hence helping to isolate their independent effects). Although REH and SV are both correlated with average summertime *T_max_* (*r* = −0.54 and *r* = 0.62, respectively), the *change* in these variables across the two decades has low correlation with *T_max_* (*r* = −0.21 and *r* = 0.05, respectively), making it a useful way to disentangle the potential independent effects on heat mortality. We ranked the cities by changes across the decades in REH and SV and then fit separate models to compare the heat-mortality relationships between cities in the top and bottom quintiles of each change metric.

#### Relative extreme heat

To quantify the change in early-season extreme heat, we adapted a measure of REH from the approach of Sheridan and Lee^25^ and Nairn and Fawcett,^26^ summarized here. First, for every *i*th day of the 5 months comprising a city’s warm season, we calculated the 92.5th percentile of *T_max_* for the period from day *i*-7 to day *i* +7 in the climatological period from 1975 to 2004 (the 30 years centered between the two decadal periods of analysis in this article). The “excess heat” for each day (*EH*_*i*_) was calculated as either the difference between that day’s *T_max_* and the climatological 92.5th percentile *T_max_* for that day or zero, whichever is larger (Equation 1):


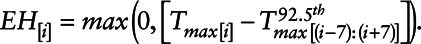
(1)

We then applied an acclimatization term to account for the intraseasonal variability in the health effects of heat: for every *i*th day, the average *T_max_* of the prior thirty days was subtracted from the *T_max_* of day *i*, and the excess heat acclimatization term (*EH*_*acc*_) was given as the greater of that value or one (Equation 2):


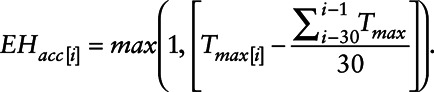
(2)

The final REH value was given as the product of these two variables (Equation 3). The REH is therefore greater for days with temperatures above the climatological 92.5th percentile *T_max_* for the 2-week period centered on that day and that are substantially warmer than the month prior (hence, REH tends to be greater in the early season).


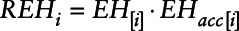
(3)

These calculations differ from those of Sheridan and Lee^25^ by using single-day values of *T_max_* instead of mean apparent temperatures averaged over three consecutive days. In addition, the raw REH values were used in the analyses rather than calculating events of REH exceedances, as was done in Sheridan and Lee.^25^

#### Social vulnerability

We calculated county-level SV by summing the counts from the 1980 and 2000 Censuses within each of the categories identified in Sociodemographic data (scaled to have a common population denominator, if applicable) and dividing by the total population—the resultant value represents the number of person-vulnerabilities per capita. We chose to calculate person-vulnerabilities per capita instead of proportion of vulnerable individuals because: (1) the census variables of interest do not contain sufficient cross-tabulation to determine discrete individuals who do not overlap multiple categories, and (2) we wanted the SV metric to reflect our assumption that individuals with multiple indicators of vulnerability (e.g., an elderly person living alone who also has an income below the federal poverty line) contributes more to the population-scale vulnerability than an individual with only one such characteristic. This framework requires an assumption of equal, additive effects of each characteristic to overall population-scale SV.^7^ Similar to relative-extreme heat, we considered only the change in SV between the two decades and grouped the cities into top and bottom quintiles of changing SV (ΔSV).

### Estimating heat-mortality relationships

We used a well-established method of time series regression analyses to estimate the association between exposure to ambient *T_max_* and the RR of death during each of the two decades (1973–1982 and 1997–2006) at the national and regional scales for the early, late, and overall warm seasons. Specifically, we used distributed-lag nonlinear models (DLNM)^27^ employing quasi-Poisson distributions to model the relation between short-term variability in *T_max_* and the RR of death from any nonexternal cause through up to 10 days of lag. We chose ten days of lag to account for potential short-term mortality displacement, consistent with the modeling choices made in other analyses,^14^ and we used sensitivity analyses to confirm that the 10-day lag provided robust, if somewhat more conservative, estimates when compared to shorter lags of 3 and 5 days. Throughout most of our analysis, we use the term “extreme-heat mortality” to refer to the RR of all-cause mortality at the 99th percentile of *T_max_* compared with the minimum-mortality temperature (MMT) of the respective decade (bounded by the 10th and 90th percentiles of *T_max_*). All statistical analyses were conducted using the *dlnm*^28^ and *mvmeta*^29^ packages in the R statistical computing environment,^30^ and all estimates are expressed with 95% confidence intervals.

Our analysis largely replicated the methods and modeling choices made in Gasparrini et al.,^14^ summarized here. First, we fit DLNMs for each city to assess the relation between daily *T_max_* and mortality over the warm season, controlling for day of week. In addition to the overall warm-season relationships, we fit separate models for early and late warm season functions using interaction terms on day of season (centered on 21 days from the first and last days of the warm season, respectively). Natural cubic splines with four degrees of freedom for seasonality and one degree of freedom per decade were applied to flexibly account for intra- and interannual variability, respectively. We placed knots at the city-specific minimum, maximum, and 75th percentile *T_max_* values. Second, we meta-analyzed these coefficients, controlling for city-level average *T_max_* to account for differential exposure-response functions by baseline climate. Finally, we ran the DLNMs again with city-specific, recentered values based on predicted pooled coefficients. We applied these modeling methods separately at the national scale (N = 186) and for each region (Northeast [N = 40], Midwest [N = 45], South [N = 66], and West [N = 35]). To test for differences beyond geographic location, we fit separate models for the top and bottom quintiles of two additional variables, as described in the previous section (see Calculating additional metrics). We used sensitivity analyses to validate the use of MMT as a referent temperature (eTables 2 and 3; http://links.lww.com/EE/A120): since our analysis is limited to the warm season—where we expect virtually no cold-related mortality—the choice of referent temperature has little material influence on the RRs at the 99th percentile.

## Results

A total of 3,810,545 nonaccidental deaths occurred during the warm-season months of 1973–1982 in the 186 metropolitan areas included in the study. The average daily maximum temperature over this period was 81.9 °F (27.7 °C) and the average 99th percentile *T_max_* across the cities was 95.8 °F (35.4 °C). Nonaccidental deaths in the latter decade (1997–2006) totaled 4,644,881, while the mean and 99th percentile *T_max_* rose to 82.8 °F (28.2 °C) and 96.7 °F (35.9 °C), respectively.

### National scale

**Figure 2.**
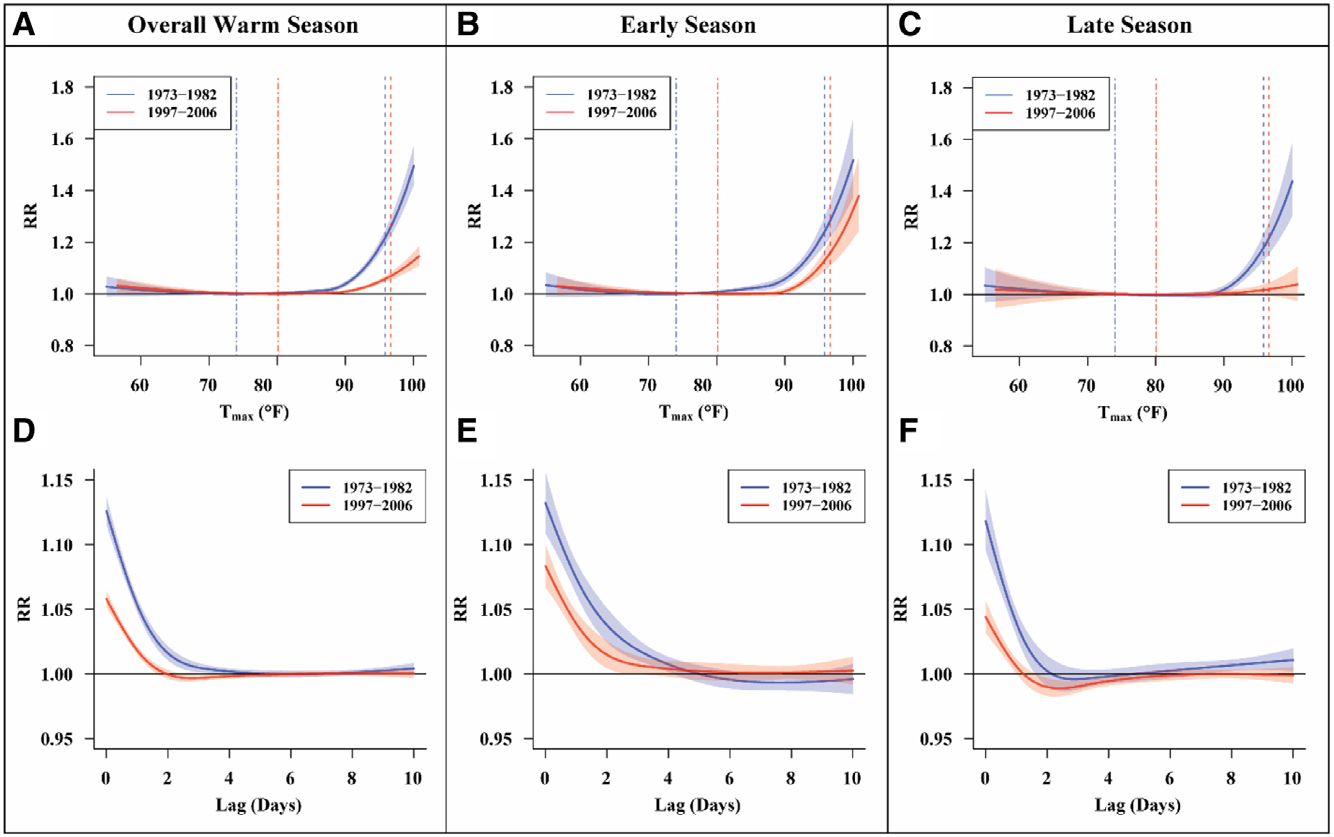
Cumulative association between ambient maximum temperature (*T_max_*) up to ten days of lag and all-cause mortality for the time periods of 1973–1982 (blue) and 1997–2006 (red) for 186 populous metropolitan areas in the contiguous United States for the overall warm season (A), early warm season (B), and late warm season (C). Dashed lines indicate the 99th percentile of *T_max_* and dash-dot lines represent the MMT, against which the other temperatures are compared to get RR. The change in RR between same-day *T_max_* (lag = 0 days) and up to 10 days later are given for the overall warm season (D), early warm season (E), and late warm season (F).

Nationally, during the warm seasons of the 1973–1982 decade, the RR of death at the 99th percentile of *T_max_* compared with the MMT (hereafter “extreme-heat mortality”; eTable 1; http://links.lww.com/EE/A120 for MMTs) was 1.22 (95% confidence interval = 1.19, 1.24). This value decreased to a RR of 1.07 (1.05, 1.08) during the 1997–2006 warm seasons (Figure 2A and Table 1). This change represents an approximately 68% decrease in the average RR across approximately 30 years.

**Table 1.** RRs of all-cause mortality from exposure to ambient maximum temperature (*T_max_*) cumulatively up to ten days of lag for the time periods of 1973–1982 and 1997–2006 during the full warm season, early warm season, and late warm season.

Region	Overall warm season RR		Early warm season RR		Late warm season RR	
	1973–1982	1997–2006	1973–1982	1997–2006	1973–1982	1997–2006
National	1.22 (1.19–1.24)	1.07 (1.05–1.08)	1.24 (1.19–1.29)	1.16 (1.11–1.21)	1.18 (1.13–1.23)	1.02 (0.99–1.05)
Northeast	1.31 (1.24–1.38)	1.11 (1.07–1.15)	1.33 (1.23–1.44)	1.32 (1.16–1.51)	1.27 (1.18–1.36)	1.00 (0.95–1.07)
Midwest	1.24 (1.19–1.29)	1.08 (1.05–1.11)	1.30 (1.15–1.47)	1.15 (1.03–1.27)	1.18 (1.08–1.29)	1.05 (0.99–1.11)
South	1.16 (1.12–1.21)	1.04 (1.02–1.07)	1.23 (1.11–1.36)	1.09 (1.00–1.18)	1.11 (1.02–1.21)	1.01 (0.97–1.06)
West	1.21 (1.16–1.25)	1.07 (1.04–1.10)	1.23 (1.13–1.35)	1.15 (1.06–1.24)	1.19 (1.08–1.32)	1.04 (0.97–1.11)

RRs are at the location-specific 99th percentile *T_max_* compared to MMT. Regions refer to US Census regions and are displayed in Figure 1.

This reduction in risk varied depending on timing within the warm season. Specifically, during the late warm season, the RR of death associated with extreme heat declined sharply from 1.18 (1.13, 1.23) in 1973–1982 to an RR of 1.02 (0.99, 1.05) in 1997–2006, while during the early warm season, the RR only declined from 1.24 (1.19, 1.29) to 1.16 (1.11, 1.21) (Figure 2B and C). There is some evidence suggesting possible differences in the exposure lag function by time of season between the two decades, though the differences are small (Figure 2D–F).

### Regional scale

Susceptibility to extreme heat varied across the four US Census regions, with the highest RR of death associated with extreme heat evident in the Northeast across the warm season in both decades, in the early phase of the warm season in both decades, and in the late phase of the warm season in the 1973–1982 decade (Table 1). Overall extreme-heat mortality decreased by similar amounts in all regions, with decreases in the RR ranging from 65% in the Northeast to 75% in the South. All regions also experienced substantial declines in late-season heat mortality, with reductions in the RR ranging from 72% in the Midwest to 100% in the Northeast (Table 1 and eFigure 1; http://links.lww.com/EE/A120).

However, the magnitude of the reduction in heat mortality during the early season varied across regions. Specifically, the decline in the early-season RR was most pronounced in the South (going from an RR of 1.23 [1.11, 1.36]) to an RR of 1.09 [1.00, 1.18]), modest in the West and Midwest, and virtually absent in the Northeast (RR of 1.33 [1.23, 1.44] in 1973–1982 vs. 1.32 [1.16, 1.51] in 1997–2006). The difference in the reduction in heat mortality between the early and late warm season is most pronounced in the Northeast (Table 1 and eFigure 1; http://links.lww.com/EE/A120).

### Comparison by susceptibility metrics

**Figure 3.**
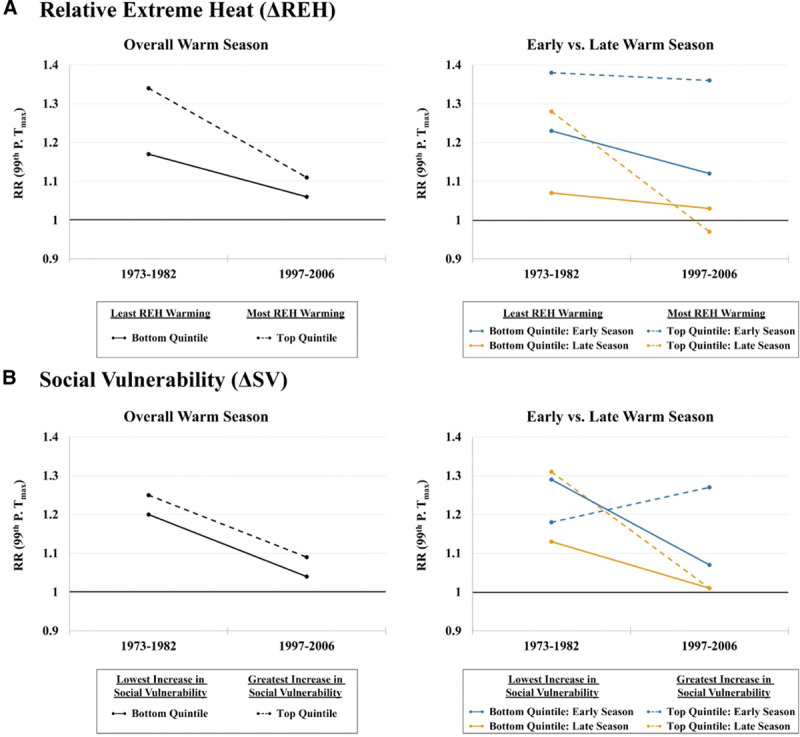
Changes in average RR of death at the 99th percentile of T_max_ relative to the minimum mortality temperature between the two decadal periods of 1973–1982 and 1997–2006 by changes in (A) REH during the early warm season and (B) proportion of socially vulnerable (SV) populations. Solid lines represent cities in the bottom quintile (least amount of REH warming and lowest increase in social vulnerability) and dashed lines represent cities in the top quintile (most amount of REH warming and greatest increase in social vulnerability). Graphs on the left are for the entire warm season (May to September), while graphs on the right show changes by early warm season (blue lines) and late warm season (orange lines). Full exposure-response functions with confidence intervals can be found in eFigure 2; http://links.lww.com/EE/A120.

We next assessed whether the magnitude of heat adaptation observed across locations was related to change over time in REH (ΔREH). We found that those cities that experienced the smallest increase in ΔREH across time (bottom quintile) saw reductions in heat mortality in both the early and late warm season. By contrast, those cities that experienced the greatest increase in ΔREH across time (top quintile) saw a pronounced decline in heat vulnerability in the late phase of the warm season but essentially no decline in heat mortality in the early warm season (Table 2 and Figure 3a; see eFigure 2a; http://links.lww.com/EE/A120, for full exposure-response functions).

**Table 2.** RRs of mortality at the 99th percentile of *T_max_* by top and bottom quintiles of changes in early-season REH and proportion of socially vulnerable (SV) populations.

Variable and quintile		Overall warm season RR		Early warm season RR		Late warm season RR	
		1973–1982	1997–2006	1973–1982	1997–2006	1973–1982	1997–2006
ΔREH	Top	1.34 (1.26–1.42)	1.11 (1.08–1.15)	1.38 (1.27–1.50)	1.36 (1.18–1.56)	1.28 (1.18–1.39)	0.97 (0.91–1.04)
	Bottom	1.17 (1.13–1.20)	1.06 (1.03–1.09)	1.23 (1.14–1.32)	1.12 (1.04–1.20)	1.07 (0.99–1.16)	1.03 (0.98–1.09)
ΔSV	Top	1.25 (1.18–1.33)	1.09 (1.06–1.12)	1.18 (1.10–1.26)	1.27 (1.13–1.44)	1.31 (1.19–1.45)	1.01 (0.96–1.06)
	Bottom	1.20 (1.13–1.27)	1.04 (1.00–1.07)	1.29 (1.10–1.52)	1.07 (0.94–1.21)	1.13 (0.98–1.31)	1.01 (0.93–1.10)

A similar pattern emerges when comparing metropolitan areas with the largest and smallest changes in the proportions of socially vulnerable subpopulations (ΔSV) between the two decades. Specifically, those cities that experienced the greatest increase in socially vulnerable populations (top quintile of ΔSV) saw a nearly 100% reduction in late-season heat mortality, but virtually no change in the early season susceptibility. By contrast, those cities with the smallest ΔSV (bottom quintile) experienced reductions in both the early- and late-season RRs (Table 2 and Figure 3B; see eFigure 2b; http://links.lww.com/EE/A120, for full exposure-response functions). Moreover, cities with the least ΔSV observed a greater decline in overall warm-season mortality.

## Discussion

The health risks posed by days of extreme heat are well documented. Although days of extreme heat are associated with lower RRs of death in the recent past compared to decades ago, it remains unknown whether this reduction in heat susceptibility varies across the months of the warm season or location. To address this knowledge gap, we compared the relationships between warm-season *T_max_* and nonexternal, all-cause mortality in 186 metropolitan areas in the conterminous United States between two decadal periods (1973–1982 and 1997–2006), with a particular focus on changes in early- versus late-warm-season mortality. As expected based on prior studies, we found substantial reductions in heat susceptibility in the more-recent decade versus our reference time point in 1973–1982. We additionally found larger reductions in heat susceptibility in the late phase of the warm season as compared to the early phase of the warm season, with substantial variation by US region.

Our finding of reduced heat susceptibility is consistent with other studies: Kinney^7^ recently summarized the literature on temporal trends in heat-related mortality, citing evidence for decreased heat susceptibility over time in New York City,^31^ the United States overall (e.g., Nordio et al.;^32^ Bobb et al.;^6^ and Davis et al.^4^), and parts of Europe (e.g., Åström et al.^33^ and Donaldson et al.^34^). However, our results suggest that these reductions in overall warm-season mortality are not uniformly distributed across the warmest months of the year. In replicating and expanding upon the methods of Gasparrini et al.,^14^ who demonstrated in a multi-country study that recent heat mortality risks tend to be greater in the early warm season at the national scale (see also Lee et al.^13^), we additionally showed that reductions between decades in heat mortality were far more pronounced in the late part of the warm season than the early part of the warm season at both the national and regional scales. Although each of the four Census regions had similar reductions in overall warm-season extreme-heat mortality, the Northeast saw virtually no reduction in early-season RR between the two decades. This observation suggests that the mechanisms by which summertime heat-related mortality is being reduced—whether through physiologic, technologic, behavioral, or infrastructural adaptations^35^—may vary both between regions and within seasons.

One common and intuitive explanation for the decline in heat mortality in recent decades is the rapid and widespread adoption of AC. However, the regional differences in the changes in early-season heat mortality observed here are not likely to be fully explained simply by improved access to AC: according to the Energy Information Administration,^36^ the Northeast, Midwest, and West had approximately the same relative increase (58%–60%) in the proportion of homes equipped with AC between 1980 and 2005. If AC were the primary causative driver of decline, then it would be expected that these regions would have seen proportional decreases in heat susceptibility over this period. Findings by Bobb et al.^6^ also support the notion that a change in AC prevalence is an insufficient explanatory factor, as they did not find a statistically significant association between increased AC prevalence and reduced heat mortality. In a more recent analysis, Sera et al.^8^ found that only 16.7% of the reduction in heat-related mortality in the United States between 1972 and 2009 was attributable to increased prevalence of AC in homes.

However, these findings are complicated by limitations in AC data, which lack the spatial and temporal specificity necessary to identify epidemiologically relevant within-county variability in AC access.^7^ Moreover, the presence of AC infrastructure itself does not necessarily imply its usage,^37^ which may be especially germane to vulnerable subpopulations who are additionally more susceptible to heat health effects (e.g., those with incomes below the federal poverty line). Assuming that increased availability of AC explains at least some of the observed reduction in heat vulnerability, the findings here of dramatically reduced late-season heat mortality but little reduction for the early season may indicate under-utilization of AC systems earlier in the summer (especially for places with greater seasonality, such as in the Northeast), either by personal choice or by managed heating, ventilation, and air conditioning systems that do not “switch over” to AC until a particular date.

This possibility of health-protective interventions being inconsistently applicable or beneficial to populations on the basis of SV is also supported in the data we presented here. Places where the population proportions of socially vulnerable groups have increased the most experienced a slight increase in early-season extreme-heat mortality over the two decades, while the risk was substantially reduced for the cities that saw declining (or marginally increasing) proportions. This concurs with other analyses that have found higher risk of heat mortality based on SV.^10,11,38^ Although there is evidence for greater SV in the southern US,^39^ assessing the change in SV (ΔSV)—which is not significantly associated with mean warm-season *T_max_*—helps disentangle these sociodemographic drivers of heat mortality from collinearity with latitude, heat, and baseline SV.

Perhaps compounding the under-utilization of protective measures such as AC is the observation that REH in the early season has increased more in some places than others between these decades, which may strain the adaptive capacity both for intra- and interannual acclimatization. Our results support this hypothesis: early-season heat mortality declined in places with reductions or little-to-no REH warming but stayed constant in the places with the greatest increase. This is consistent with expectations based on the underlying principle of within-season acclimatization: given that populations are more susceptible to heat earlier in the season before they have had a chance to acclimate to the warming weather,^13,14^ it follows that increasingly extreme temperatures during this early time period may further stress the population’s coping abilities and leave them at an elevated risk.

These results have implications in the context of recent and future climate change. Although others have argued that heat adaptation has outpaced the rate of recent warming and mean temperatures (e.g., Christidis et al.^40^ and Kinney^7^), they may not have considered within-season variability or more-specific indicators of climate change, such as changes in the occurrence of REH. This is particularly important since North America has experienced earlier onsets of spring driven by seasonal warming^41–43^ and an increasing trend in the annual number of REH events.^25^

Findings from this study suggest that the benefits of heat-health adaptations have not been consistently or fully realized throughout the warm season, and that they vary by geographic region and by the magnitude of changes in early-season REH and indicators of SV. In other words, our results suggest that there may be an important opportunity to further reduce susceptibility to days of extreme heat by focusing adaptation efforts on the early part of the warm season. Bolstering location-specific heat adaptations and ensuring efficacy across the entire population should be a priority for local and regional public health practitioners and decision-makers.

This analysis was limited by the period of available mortality data (extending only through 2006), which precluded the ability to assess whether the early-season RRs identified here persist in the present day. Additionally, the lack of specificity of the cause of death in the mortality data and a lack of morbidity data (e.g., hospital admissions) prevented assessments on specific pathways between heat and health outcomes that could be useful for informing more-targeted health interventions. Finally, the study design required the use of consistent months to define the warm season between the two periods, which constrained the ability to capture intra-annual warming variability sufficient to alter the identification of the warmest months of the year; however, using 5 months instead of four mitigated this in all but two cities, introducing an amount of error assumed to be negligible.

## Conclusions

Although the RR of mortality on days of extreme heat decreased substantially between 1973 and 2006 in the United States, considerable risk remains. Within-season variability in the heat-mortality relation appears to have increased over this period, with much greater reductions in late-season heat mortality than in the early season. This divergence is most pronounced in the Northeast region, in places with greater increases in early-season REH, and in cities with greater increases in the proportion of socially vulnerable populations. Substantial heat mortality risks therefore remain in the United States, especially during the early warm season. Adaptation measures to reduce the public health burden of extreme heat should be assessed for efficacy during the early warm season to maximize their protective effects.

## Conflicts of interest statement

G.A.W. has received consulting income from the Health Effects Institute (Boston, MA) and recently served as a paid visiting scientist at Google, LLC (Mountain View, CA). The other author declare that they have no competing conflicts of interest with respect to the content of this manuscript.

## ACKNOWLEDGMENTS

We gratefully acknowledge Drs. Kate Weinberger and Melissa Eliot for their data and programming support.

## Supplementary Material


