# Supplementary material for: Spatial and intraseasonal variation in changing susceptibility to extreme heat in the United States
Source: Environ Epidemiol. 2021 Feb 26;5(2):e136. doi: 10.1097/EE9.0000000000000136 (PMC8043727; doi:10.1097/EE9.0000000000000136)
Supplement: Supplementary file 1 [file ee9-5-e136-s001.docx]

**Supplemental Digital Content for**

***Spatial and Intra-seasonal Variation in Changing Susceptibility to Extreme Heat in the United States***

Keith R. Spangler^1,2,3,4^ and Gregory A. Wellenius^1, 3^

1 Boston University School of Public Health, Department of Environmental Health, Boston, MA, USA

2 Brown University Department of Earth, Environmental, and Planetary Sciences, Providence, RI, USA

3 Brown University School of Public Health, Department of Epidemiology, Providence, RI, USA

4 Institute at Brown for Environment and Society, Brown University, Providence, RI, USA

Corresponding author: Keith R. Spangler ([**krspangl@bu.edu**](mailto:krspangl@bu.edu))

715 Albany St., T4W, Boston, MA 02118-2526

| eTable 1. Minimum-mortality temperatures (MMT) |  | 1 |
| --- | --- | --- |
|  |  |  |
| eTable 2. Relative risks compared to 10^th^ percentile *T_max_* |  | 2 |
|  |  |  |
| eTable 3. Relative risks compared to 50^th^ percentile *T_max_* |  | 2 |
|  |  |  |
| eFigure 1. Exposure-response functions by Census region |  | 3 |
|  |  |  |
| eFigure 2. Exposure-response functions by ∆SV and ∆REH |  | 4 |
|  |  |  |

| **Group of Cities** | **Minimum-Mortality**  **Temperature (MMT)** | | **Minimum Mortality**  **Percentile (MMP)** | |
| --- | --- | --- | --- | --- |
|  | 1973-1982 | 1997-2006 | 1973-1982 | 1997-2006 |
| National | 74.0 ˚F | 80.1 ˚F | 15^th^ | 32^nd^ |
| Northeast | 74.2 ˚F | 74.8 ˚F | 34^th^ | 32^nd^ |
| Midwest | 72.5 ˚F | 86.0 ˚F | 22^nd^ | 77^th^ |
| South | 79.2 ˚F | 83.7 ˚F | 10^th^ | 22^nd^ |
| West | 81.5 ˚F | 72.6 ˚F | 44^th^ | 10^th^ |
| ∆REH Top Quartile | 66.7 ˚F | 68.7 ˚F | 10^th^ | 12^th^ |
| ∆REH Bottom Quartile | 80.0 ˚F | 78.2 ˚F | 28^th^ | 18^th^ |
| ∆SV Top Quartile | 81.2 ˚F | 81.5 ˚F | 35^th^ | 31^st^ |
| ∆SV Bottom Quartile | 73.9 ˚F | 85.0 ˚F | 10^th^ | 52^nd^ |

**eTable 1**: The minimum-mortality temperatures (MMT) and percentiles (MMP) used in each of the distributed-lag nonlinear

models estimating the relative risk of all-cause, non-external mortality in various groups of U.S. cities.

| **Referent Temperature Set at 10^th^ Percentile of *T_max_*** | | | | | | |
| --- | --- | --- | --- | --- | --- | --- |
| **Group of Cities** | **Overall Warm Season RR** | | **Early-Season RR** | | **Late-Season RR** | |
|  | **1973-1982** | **1997-2006** | **1973-1982** | **1997-2006** | **1973-1982** | **1997-2006** |
| **National** | 1.22  (1.19,1.24) | 1.07 (1.05,1.08) | 1.24  (1.19,1.30) | 1.16 (1.10,1.21) | 1.18 (1.13,1.23) | 1.02 (0.99,1.05) |
| **Northeast** | 1.31  (1.24, 1.38) | 1.10  (1.06, 1.14) | 1.33  (1.23, 1.44) | 1.31  (1.16, 1.49) | 1.24  (1.15, 1.34) | 0.99  (0.93, 1.07) |
| **Midwest** | 1.23  (1.18, 1.29) | 1.06  (1.02, 1.09) | 1.30  (1.15, 1.46) | 1.10  (1.00, 1.21) | 1.17  (1.07, 1.28) | 1.04  (0.97, 1.10) |
| **South** | 1.16  (1.12, 1.21) | 1.04  (1.02, 1.07) | 1.23  (1.11, 1.36) | 1.09  (1.00, 1.18) | 1.11  (1.02, 1.21) | 1.01  (0.97, 1.06) |
| **West** | 1.20  (1.15, 1.25) | 1.07  (1.04, 1.10) | 1.23  (1.13, 1.35) | 1.15  (1.06, 1.24) | 1.17  (1.07, 1.27) | 1.04  (0.97, 1.11) |
| **∆REH Q5** | 1.34  (1.26, 1.42) | 1.11  (1.07, 1.15) | 1.38  (1.27, 1.50) | 1.36  (1.18, 1.56) | 1.28  (1.18, 1.39) | 0.97  (0.91, 1.04) |
| **∆REH Q1** | 1.16  (1.12, 1.2) | 1.06  (1.03, 1.09) | 1.22  (1.13, 1.31) | 1.11  (1.04, 1.20) | 1.07  (0.99, 1.15) | 1.04  (0.99, 1.10) |
| **∆SV Q5** | 1.24  (1.17, 1.32) | 1.09  (1.05, 1.12) | 1.17  (1.10, 1.26) | 1.27  (1.13, 1.43) | 1.30  (1.18, 1.43) | 1.01  (0.95, 1.06) |
| **∆SV Q1** | 1.20  (1.13, 1.27) | 1.03  (0.99, 1.07) | 1.29  (1.10, 1.52) | 1.07  (0.94, 1.21) | 1.13  (0.98, 1.31) | 0.99  (0.90, 1.08) |

**eTable 2**: Relative risks of all-cause, non-external mortalities at the 99^th^ percentile of *T_max_* compared to the 10^th^ percentile of *T_max_* (as opposed to the minimum-mortality temperature used in the main text). Top quintile is “Q5” and bottom is “Q1.”

| **Referent Temperature Set at 50^th^ Percentile of *T_max_*** | | | | | | |
| --- | --- | --- | --- | --- | --- | --- |
| **Group of Cities** | **Overall Warm Season RR** | | **Early-Season RR** | | **Late-Season RR** | |
|  | **1973-1982** | **1997-2006** | **1973-1982** | **1997-2006** | **1973-1982** | **1997-2006** |
| **National** | 1.21  (1.18,1.24) | 1.07 (1.05,1.08) | 1.22  (1.17,1.28) | 1.16 (1.11,1.21) | 1.18 (1.13,1.24) | 1.02 (0.99,1.05) |
| **Northeast** | 1.31  (1.24, 1.38) | 1.11  (1.07, 1.15) | 1.33  (1.22, 1.44) | 1.32  (1.16, 1.51) | 1.27  (1.18, 1.36) | 1.00  (0.94, 1.07) |
| **Midwest** | 1.23  (1.18, 1.28) | 1.08  (1.04, 1.11) | 1.28  (1.13, 1.44) | 1.13  (1.02, 1.25) | 1.18  (1.08, 1.29) | 1.05  (0.99, 1.11) |
| **South** | 1.14  (1.09, 1.18) | 1.04  (1.02, 1.06) | 1.18  (1.07, 1.31) | 1.09  (1.00, 1.18) | 1.09  (1.00, 1.19) | 1.01  (0.97, 1.06) |
| **West** | 1.21  (1.16, 1.25) | 1.06  (1.03, 1.09) | 1.24  (1.13, 1.35) | 1.14  (1.05, 1.24) | 1.19  (1.08, 1.32) | 1.03  (0.96, 1.10) |
| **∆REH Q5** | 1.33  (1.26, 1.41) | 1.10  (1.07, 1.14) | 1.37  (1.25, 1.49) | 1.35  (1.17, 1.56) | 1.31  (1.21, 1.41) | 0.98  (0.92, 1.04) |
| **∆REH Q1** | 1.17  (1.13, 1.20) | 1.06  (1.03, 1.09) | 1.22  (1.14, 1.32) | 1.12  (1.04, 1.2) | 1.07  (0.98, 1.16) | 1.02  (0.97, 1.08) |
| **∆SV Q5** | 1.25  (1.18, 1.33) | 1.09  (1.05, 1.12) | 1.17  (1.09, 1.26) | 1.28  (1.13, 1.45) | 1.32  (1.20, 1.45) | 1.01  (0.96, 1.06) |
| **∆SV Q1** | 1.16  (1.10, 1.24) | 1.04  (1.00, 1.07) | 1.27  (1.08, 1.48) | 1.07  (0.94, 1.21) | 1.07  (0.92, 1.25) | 1.01  (0.93, 1.10) |

**eTable 3**: Relative risks of all-cause, non-external mortalities at the 99^th^ percentile of *T_max_* compared to the 50^th^ percentile of *T_max_* (as opposed to the minimum-mortality temperature used in the main text). Top quintile is “Q5” and bottom is “Q1.”


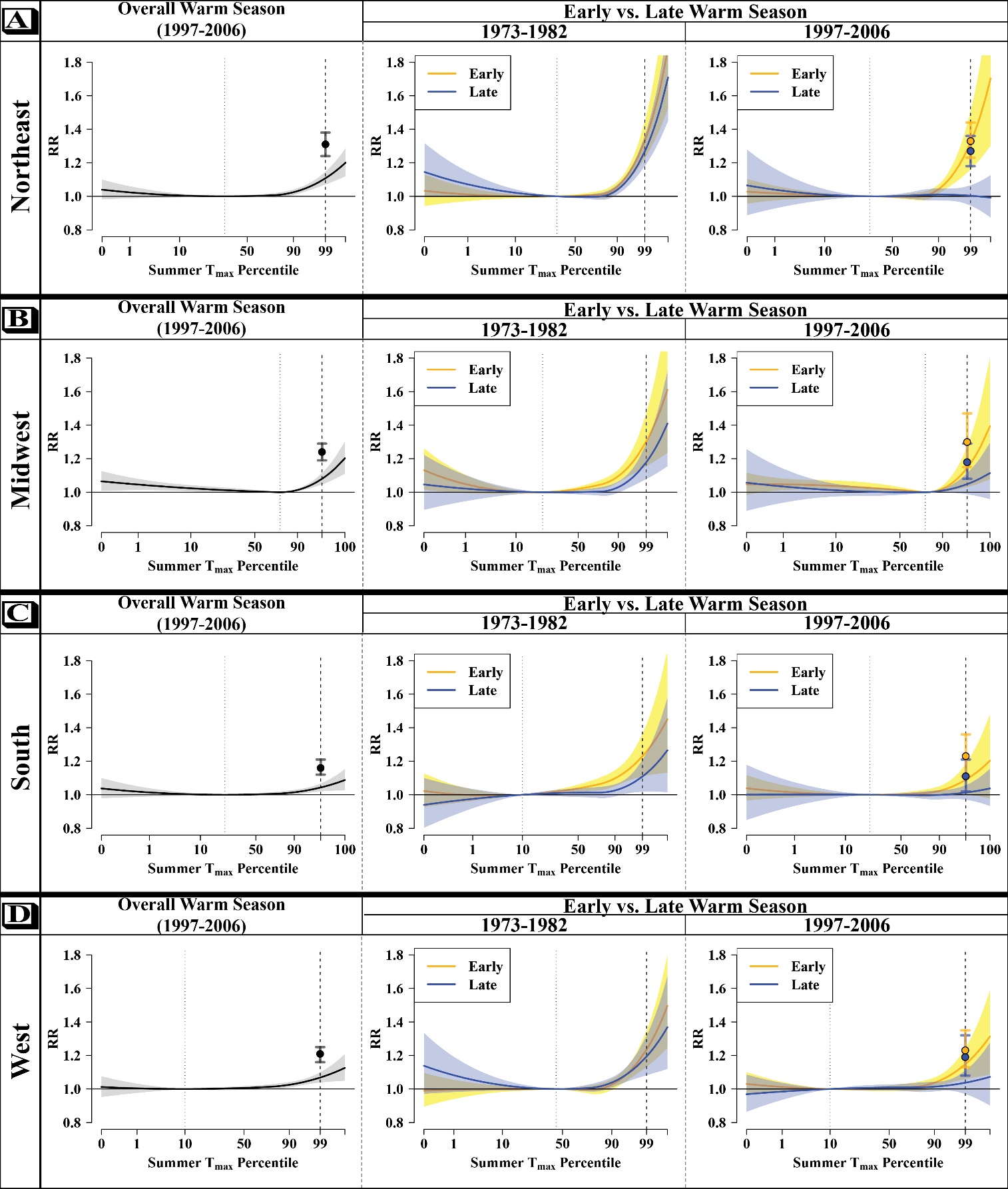


**eFigure 1**: Associations between T_max_ and relative risk (RR) of mortality by Census region of the US. The first column shows the heat-mortality relationship for the entire warm season for 1997-2006 (dots denote the RR with confidence intervals for 1973-1982 for comparison). Heat-mortality associations during the early (yellow) and late (blue) warm seasons are given for 1973-1982 (second column) and 1997-2006 (third column). Yellow and blue dots are placed on the 99^th^ percentile in the third column corresponding to the RR from the earlier decade (shown in the second column).


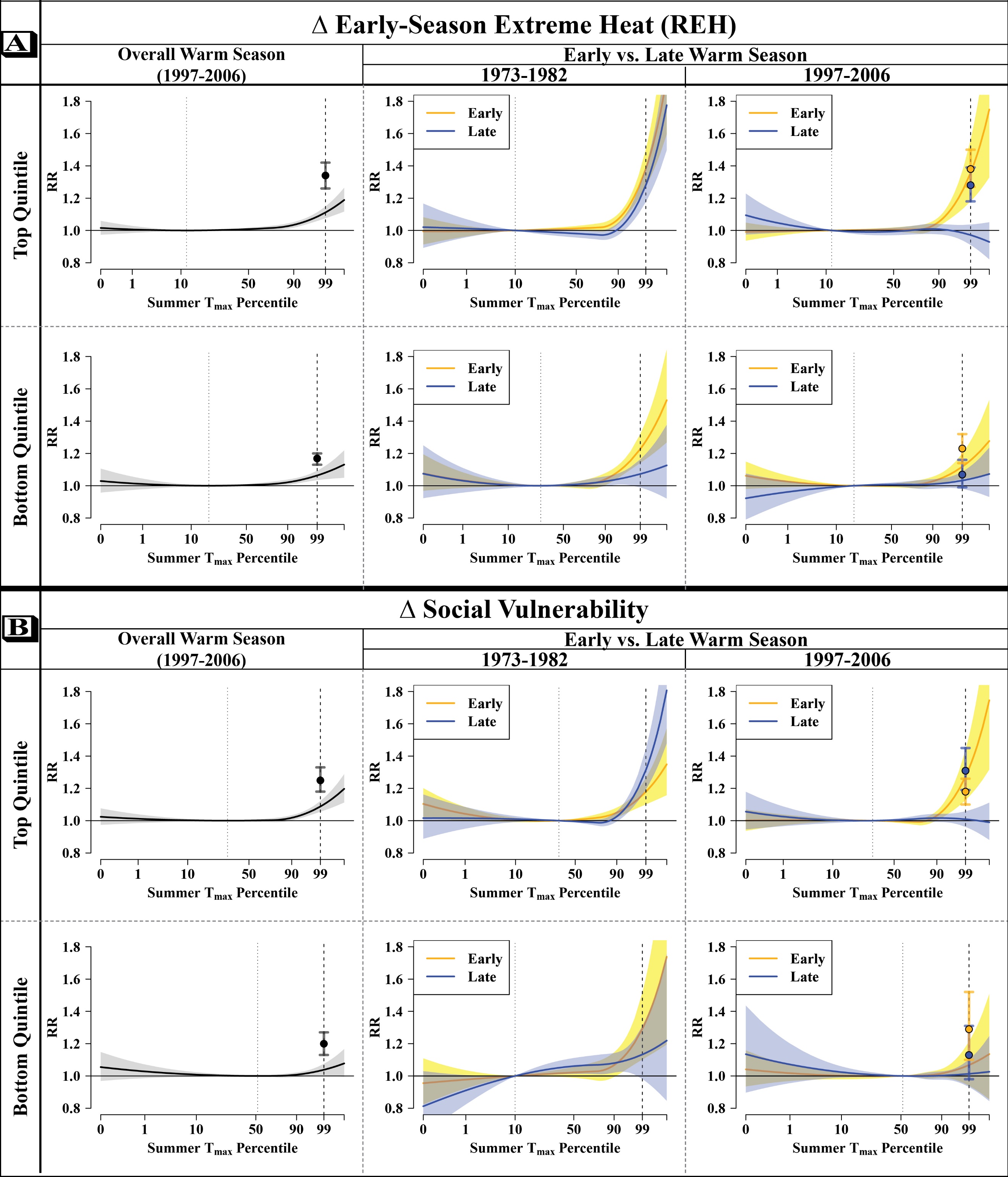


**eFigure 2**: Associations between T_max_ and cumulative relative risk (RR) of mortality between top and bottom quintiles of: (a) change in early-season relative extreme heat (REH) and (b) change in proportions of vulnerable populations. The first column shows the heat-mortality relationship for the entire warm season for 1997-2006 (dots denote the RR with confidence intervals for 1973-1982). Heat mortality during the early (yellow) and late (blue) seasons are given for 1973-1982 (second column) and 1997-2006 (third column). Yellow and blue dots are placed on the 99^th^ percentile in the third column corresponding to the RR from the earlier decade. Thin dotted lines indicate minimum-mortality temperatures (MMT) and dashed lines represent 99^th^ percentile T_max_.
